# Fatty fish intake and cognitive function: FINS-KIDS, a randomized controlled trial in preschool children

**DOI:** 10.1186/s12916-018-1020-z

**Published:** 2018-03-12

**Authors:** Jannike Øyen, Ingrid Kvestad, Lisa Kolden Midtbø, Ingvild Eide Graff, Mari Hysing, Kjell Morten Stormark, Maria Wik Markhus, Valborg Baste, Livar Frøyland, Berthold Koletzko, Hans Demmelmair, Lisbeth Dahl, Øyvind Lie, Marian Kjellevold

**Affiliations:** 1 Institute of Marine Research (IMR), P.O Box 1870 Nordnes, NO-5817 Bergen, Norway; 2 Regional Centre for Child and Youth Mental Health, Uni Research Health, Bergen, Norway; 3 Department of Health Promotion and Development, University of Bergen, Bergen, Norway; 4 Uni Research Health, Bergen, Norway; 5 Ludwig-Maximilians-Universität München, Dr. von Hauner Children’s Hospital, Div. Metabolic & Nutritional Medicine, University of Munich Medical Center, Munich, Germany

**Keywords:** Cognitive function, Docosahexaenoic acid, Eicosapentaenoic acid, Fatty fish, Omega-3 index, Vitamin D, Preschoolers, Wechsler Preschool and Primary Scale of Intelligence, 9-Hole Peg Test

## Abstract

**Background:**

Marine resources including fatty fish are important sources of n-3 long chain polyunsaturated fatty acids (n-3 LC-PUFAs), which are important for brain development. To our knowledge, this is the first randomized controlled trial (RCT) investigating the impact of fatty fish on cognition in preschool children. The purpose of the trial was to investigate whether an increased intake of fatty fish compared to meat improves cognitive function in children 4–6 years old.

**Methods:**

The children (*n* = 232) in this two-armed RCT, Fish Intervention Studies-KIDS (FINS-KIDS) were recruited from 13 kindergartens in Bergen, Norway. They were randomly assigned to lunch meals with fatty fish (herring/mackerel) or meat (chicken/lamb/beef) three times a week for 16 weeks. The fish and meat were weighed before and after the meals to record the exact consumption (dietary compliance). The primary outcome was cognitive function measured by the Wechsler Preschool and Primary Scale of Intelligence, 3rd edition (WPPSI-III) and fine-motor coordination measured by the 9-Hole Peg Test (9-HPT) at pre- and post-intervention. Biological samples (blood, urine, hair), and questionnaires to the caregivers were included at both time points. Linear mixed effect models with a random intercept for kindergarten were used to analyze changes from pre- to post-intervention in the primary outcome variables.

**Results:**

There were 218 children included in the trial (105 in the fish, and 113 in the meat group). The children consumed a mean (standard deviation) of 2070 (978) g fish or 2675 (850) g meat from the study meals (*p* < 0.0001). The fish group had a significant increase of red blood cell n-3 LC-PUFAs. The intervention had no effect on the WPPSI-III scores (mean change total raw score; fish group 17.7, 95% confidence interval (CI) 14.8–20.7 vs meat group 17.8, 95% CI 15.0–20.6, *p* = 0.97) in the main analyses. In the sub-analyses, adjusting for dietary compliance, the fish group showed a higher improvement on total raw score (20.4, 95% CI 17.5–23.3) compared to the meat group (15.2, 95% CI 12.4–18.0, *p* = 0.0060); docosahexaenoic acid mediated this effect.

**Conclusions:**

There was no beneficial effect of fatty fish compared to meat on cognitive functioning in the preschool children. When considering dietary compliance, we found a beneficial effect of fatty fish on cognitive scores.

**Trial registration:**

ClinicalTrials.gov, NCT02331667 December 17, 2014.

**Electronic supplementary material:**

The online version of this article (10.1186/s12916-018-1020-z) contains supplementary material, which is available to authorized users.

## Background

Fish are important sources of n-3 long chain polyunsaturated fatty acids (n-3 LC-PUFAs). Eicosapentaenoic acid (EPA) and docosahexaenoic acid (DHA) play a critical role in optimizing brain development [1, 2] and may therefore be important for cognitive functioning from childhood throughout the lifespan [3, 4].

Intervention studies examining the effect of n-3 LC-PUFA supplementation on cognitive function in children have shown both positive [5–8] and null results [6, 9, 10]. A meta-analysis of randomized controlled intervention trials (RCTs) concluded that there is a beneficial effect of n-3 LC-PUFA supplementation on cognitive development in infants, but that there is no consistent evidence for a similar effect in later childhood [11]. Due to the high concentration of n-3 LC-PUFA in fatty fish, an effect of fatty fish consumption on cognitive function seems plausible. In addition to n-3 LC-PUFAs, fish contains micronutrients such as vitamin D and iodine that may have positive influences on cognition in infants and children [12–15], and the differences in bioavailability of n-3 LC-PUFAs between fish meals and supplements may give superiority effects for fish intake [16]. Observational studies have shown a positive relation between fish consumption and cognitive function in adolescents and adults [17–19], with findings suggesting that higher intakes of seafood are associated with higher performance on cognitive tests [20]. To our knowledge, there are no RCTs examining fatty fish consumption and cognitive function in children.

The purpose of this RCT was to investigate if increased intake of EPA- and DHA-rich herring and mackerel improve cognitive functions, compared to meat, in terms of both general intellectual abilities and intellectual functioning in the verbal and performance domains and processing speed in 4- to 6-year-old preschool children.

## Methods

### Trial design and oversight

In the two-armed Fish Intervention Studies-KIDS (FINS-KIDS), conducted in Bergen, Norway, preschool children were individually randomized to receive three hot lunch meals per week containing fatty fish or meat for 16 weeks.

The trial has approval from the Regional Committees for Medical and Health Research Ethics North (2014/1396). Written informed consent was obtained from the participants’ caregivers. Participants could withdraw from the study at any time without giving any reason.

### Enrollment and randomization

Seventeen out of the total 250 kindergartens in Bergen municipality were invited, and 13 agreed to participate. Invitations were sent out to kindergartens in different districts to ensure participants with different socioeconomic status. Children 4–6 years old, with sufficient understanding of the Norwegian language to undergo cognitive testing, and whose caregivers had sufficient language skills to answer online questionnaires in Norwegian, were included. Exclusion criteria were any known food allergies. Children were randomly assigned in a 1:1 ratio to receive lunch meals with either fatty fish or meat, stratified on gender. A blinded researcher generated independent allocation sequences and the randomization lists for each kindergarten, using Microsoft Excel. Another researcher controlled the randomization procedure.

### Procedure

The trial took place between January and June 2015. The inclusion and pre-intervention tests were done during a 6-week period. The intervention started within 1 week after the pre-intervention data were collected, and the post-intervention testing started within 1 week after the last study meal was consumed. Pre- and post-intervention testing included cognitive tests, blood, urine, and hair sampling, and online questionnaires to the caregivers. A catering company (Søtt+Salt A/S, Bergen) prepared and delivered the study meals to each kindergarten. Each meal contained 50–80 g fatty fish (herring/mackerel) or meat (chicken/lamb/beef). Meat was chosen as the comparison to the intervention (fatty fish) to enable control of the intake in the comparison group and to ensure that the supply of nutrient was different between the two groups. A variety of identical side dishes was provided for both intervention groups. Results from the analyses of energy, nutrients, and undesirable substances in the meat and fish from the study meals are presented in Additional file 1: Table S1.

Research assistants, not otherwise involved in the study, served the meals and weighed the fish and meat with identical digital weights (Digital Glass Kitchen Scale, Soehnle, Nassau, Germany) before and after the lunch. The exact consumption of each meal in grams was summed up to a total amount, constituting dietary compliance for each child. The research assistants recorded when children were absent and when they were present during the meals to secure that the children ate from their own meal only.

### Outcome measures

#### Cognitive tests

The primary outcome was cognitive function measured by the general intellectual ability test Wechsler Preschool and Primary Scale of Intelligence, 3rd edition (WPPSI-III) [21] and the 9-Hole Peg Test (9-HPT) [22]. WPPSI-III is a widely used test to measure general intellectual abilities [23] which has been translated and standardized to a Norwegian population and has Norwegian norms [24]. The evaluation of the Norwegian version demonstrates good psychometric properties, showing that this tool is a reliable and valid measure of intellectual abilities in the Norwegian population. We used eight sub-tests (Information, Vocabulary, Word Reasoning, Block Design, Matrix Reasoning, Picture Concepts, Coding, and Symbol Search) to generate a Full-Scale IQ (FIQ), Verbal IQ (VIQ), Performance IQ (PIQ), and a Processing Speed Quotient (PSQ). Both age-corrected raw scores and scaled scores were used in the analyses. The 9-HPT is a validated test of dexterity and fine-motor coordination in children where the time required for task completion is recorded for both the dominant and non-dominant hand [22, 25].

Nine test administrators (students in medicine and nutrition) were trained for 30 h on administering the WPPSI-III and 9-HPT prior to the first test phase. A clinical child psychologist (IK) was responsible for the training and supervision. Ten percent of the tests were scored by two administrators, and the inter-class correlations ranged from 0.98 to 1.00, indicating high inter-rater agreement. Tests were administered in separate rooms in the kindergartens, with noise kept to a minimum. Each session lasted for 60–90 min including a small break. The testers were blinded to the treatment conditions.

All WPPSI-III forms were cross-checked and then scored by test administrators using the Pearson WPPSI-III Scoring Assistant® software. Following validation, two data entry operators entered the data separately.

#### Questionnaires

A revised version of a validated food frequency questionnaire (FFQ) [26–28] was filled in online by the caregivers pre- and post-intervention to assess the food intake during the last 3 months. The caregivers were instructed not to include intervention meals. The questionnaire also included questions regarding demographics (children’s weight/height, parental education, family income, physical activity). We do not have information on whether mothers, fathers, or other caregivers filled in the questionnaires for the children.

An index calculation was carried out based on the development and validation of a seafood index by Markhus et al. [27]. These calculations converted ordinal frequency data in the FFQ to numerical scale data. The food items other than seafood were converted in a similar manner.

In addition, a diet score, as previously described by Handeland et al. [28], to assess the children’s adherence to Norwegian dietary recommendations [29] was determined from the FFQ results. The recommendations used included the following: eat at least three portions of vegetables and two portions of fruit every day, eat at least four whole-grain products every day, eat fish corresponding to two to three dinner servings a week, limit the intake of red meat products, choose low-fat dairy products, limit the intake of added sugar, choose water as the recommended beverage, and do some form of physical activity at least 30 min every day. The diet score ranged from 0 to 8, with 8 as the best adherence to the dietary recommendations. The diet score was divided into the three categories “low” (0–3 points), “moderate” (4–5 points), and “high” (6–8 points).

#### Biochemical analyses

Two biomedical scientists blinded to treatment conditions performed the blood sampling in each kindergarten. Venous blood was collected in BD Vacutainer® K2E 7.2-mg vials for preparation of red blood cells (RBC) and BD Vacutainer® SST™ II Advance for preparation of serum and centrifuged (10 min/1000 g/20 °C) within 30 min of sampling, transferred to Cryotubes (Nunc/Roskilde/Denmark), and transported on dry ice to storage at −80 °C until analysis. Mixed pre- and post-intervention samples were analyzed after the intervention.

Fatty acid composition of total RBC was determined by standardized procedures at the Institute of Marine Research (IMR) [30], using ultrafast gas chromatography (UFGC) (Thermo Electron Corporation, Massachusetts, USA).

The s-25-hydroxyvitamin D_3_ (25(OH)D)_3_ concentrations were determined by standardized procedures at IMR [31], using a liquid chromatographic-tandem mass spectrometric (LC-MS/MS) assay adding acetonitrile and an internal standard (^2^H 25OH vitamin D_3_) to the samples.

s-ferritin was analyzed at Haraldsplass Diakonale Hospital, Bergen, by an automated electrochemiluminescence immunoassay (ECLIA) on Cobas e601 (Roche).

Urinary iodine concentration (UIC) was determined in spot samples by inductive coupled plasma mass spectrometry (ICP-MS) by standardized procedures at IMR [32].

Total hair mercury concentration (THHg) was obtained by cutting a bundle of hairs approximately 3 mm in diameter as close to the scalp as possible from the occipital area. Samples were analyzed using a Direct Mercury Analyzer (DMA-80, Milestone) [33]. Human hair IAEA-086 was used as standard reference material (powder, International Atomic Energy Agency (IAEA), Vienna, Austria).

### Sample size calculations

The mean general ability score of the WPPSI-III is 100 with a standard deviation (SD) of 15 [24]. An effect size of 0.37 (corresponding to approximately 5 WPPSI-III points) can be detected with a power of 80% and a significance level of alpha = 0.05 by studying 116 subjects per group. Estimating 20% dropout, a total of 290 children ought to be invited.

### Statistical analyses

Continuous variables are expressed as mean and SD, and categorical variables as numbers and percentages. For the WPPSI-III, we use scaled scores to describe the sample (Table 1) and raw scores in the regression models and for the mediation analyses. We chose raw scores for the statistical analyses to ensure that the scores had sufficient range and variance to identify the possible impact of fatty fish on cognition [34].

A paired samples *t* test was used to compare variables within the intervention groups, and an independent samples *t* test was used for comparisons between the intervention groups. The correlation between diet score and parental education was assessed by using Pearson product moment correlation. In the main analyses, the paired samples *t* test was used to compare differences between pre- and post-intervention values in the primary outcome variables within each intervention group. Linear mixed effect models with a random intercept for kindergarten were used to analyze changes from pre- to post-intervention in the primary outcome variables. The models were adjusted for age, which is appropriate when using raw scores, and the pre-intervention score. Unadjusted analyses were also performed but did not differ from the adjusted analyses (data not shown).

In sub-analyses, we first included dietary compliance, and secondly the interaction between the intervention group and dietary compliance. To illustrate the possible interaction between intervention and dietary compliance on WPPSI-III total and sub-scale raw scores, we used scatter plots with estimated regression lines from the model. A likelihood ratio test was applied to compare models. The models were also adjusted for parental education, family income, gender, FFQ-reported fish intake (background diet), and THHg, but these adjustments did not alter the estimates for any of the models substantially (data not shown).

The potential mediation effect of biochemical parameters (change in pre- to post-intervention of RBC linoleic acid, arachidonic acid, EPA, docosapentaenoic acid, DHA, s-25(OH)D, s-ferritin, and UIC) and reported dietary intake (FFQ data) on the significant association between intervention and WPPSI-III and 9-HPT was assessed by standard methods [35]. The mediator variables were assessed one at a time and adjusted for pre-intervention score, age, and dietary compliance.

Two-tailed *p* < 0.05 was considered statistically significant. Analyses were performed using data analysis and statistical software (Stata IC 14.2) and Statistical Package for the Social Sciences (SPSS® Statistics Version 24).

## Results

### Study population

**Fig. 1:**
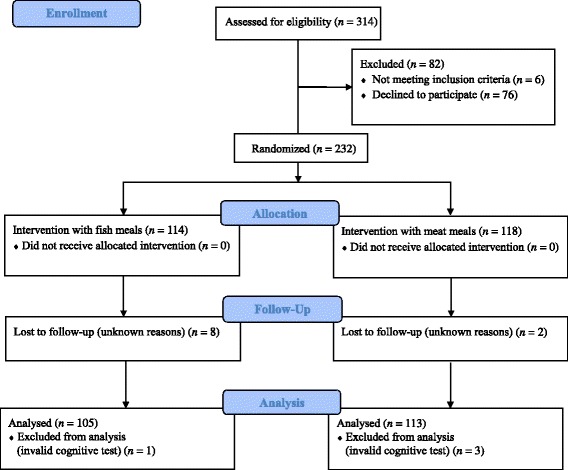
Flowchart showing the study population

In total, 314 children were eligible and invited to participate. The families of 232 children (73.9%) were enrolled and allocated for randomization between December 19, 2014 and February 9, 2015. The intervention was completed on June 12, 2015. Of the 232 children, 114 (49.1%) were randomized to fish and 118 (50.9%) to meat meals. Ten children did not complete the intervention period, and four were excluded from the analyses due to invalid cognitive tests (two due to vision problems and two because of incomplete tests due to distractibility). Thus, the final sample included 218 (93.7%) children, 105 (92.1%) and 113 (95.8%) in the fish and meat groups, respectively (Fig. 1).

The mean (SD) age of the children was 5.2 (0.6) years, and 112 (51.4%) were girls (Table 1). There were no differences between the participants in the fish and meat group, or completers and non-completers on baseline characteristics.

**Table 1:** Baseline characteristics, by intervention group

	Number, *N*	Fish group (*n* = 105)	Meat group (*n* = 113)
Demographics			
Age, years	218	5.2 (0.6)	5.2 (0.6)
Body weight, kg	178	20.0 (3.3)	20.2 (3.0)
Body height, cm	178	113.6 (5.9)	113.6 (6.5)
Boys, *n* (%)	106	52 (49.5%)	54 (47.8%)
Girls, *n* (%)	112	53 (50.5%)	59 (52.2%)
Right handedness	200	95 (90.5%)	105 (92.9%)
Left handedness	18	10 (9.5%)	8 (7.1%)
Family income in NOK^a^			
< 200,000–749,999	51	24 (27.0%)	27 (25.7%)
750,000–1,249,999	111	47 (52.8%)	64 (61.0%)
1,250,000– > 2,000,000	32	18 (20.2%)	14 (13.3%)
Education parents, years	195	15.4 (1.7)	15.4 (1.6)
WPPSI-III, scaled scores			
FIQ	218	100.2 (12.0)	98.1 (10.8)
VIQ	218	99.5 (13.5)	98.5 (12.3)
PIQ	218	100.2 (12.5)	96.9 (12.0)
PSQ	218	104.5 (12.7)	104.0 (12.0)
9-HPT, seconds			
Dominant hand	218	30.6 (7.8)	30.5 (7.6)
Non-dominant hand	218	37.0 (10.3)	36.6 (8.9)
Dietary intake from FFQ, meals/week			
Seafood as dinner	197	1.8 (0.9)	1.6 (0.9)
Mackerel as dinner	197	0.1 (0.3)	0.1 (0.3)
Herring as dinner	197	0.0 (0.1)	0.0 (0.0)
Red meat as dinner	197	2.6 (0.8)	2.4 (0.9)
Chicken as dinner	197	1.4 (0.9)	1.2 (0.9)
Fish as bread spread	197	1.4 (1.5)	1.2 (1.4)
n-3 LC-PUFA supplements	74	35 (38.5%)	39 (36.8%)
No n-3 LC-PUFA supplements	123	56 (61.5%)	67 (63.2%)

Values indicate mean (SD) or *n* (%). No significant differences were observed between the intervention groups

Abbreviations: *FFQ* food frequency questionnaire, *FIQ* Full-Scale IQ, *9-HPT* 9-Hole Peg Test, *n-3 LC-PUFA* n-3 long chain omega-3 fatty acids, *PIQ* Performance IQ, *PSQ* Processing Speed Quotient,

*VIQ* Verbal IQ, *WPPSI-III* Wechsler Preschool and Primary Scale of Intelligence, 3rd edition

^a^100 NOK = approximately 10€ or 11$

### Background diet

The background diet as reported by meals per week and as a diet score (Additional file 2: Figures S1 and S2) from the FFQ was similar in the intervention groups at baseline (Table 1 and Additional file 2: Table S2). No significant differences within or between the groups, except for a little lower intake of red meat in the fish group, were observed from pre- to post-intervention (Additional file 2: Table S2).

The results from the diet score at baseline as presented in Additional file 2: Figure S1 show the adherence to the recommendations given as percentage of children (*n* = 197). Only 3% of the children adhere to the recommendations for fruit and vegetables, whereas 49% and 50% adhered to the recommendations for fish and added sugar, respectively. The highest compliance scores were found for whole grains, red meat, dairy products, and water, with 84%, 85%, 90%, and 96%, respectively.

Additional file 2: Figure S2 shows the percentage of children (*n* = 197) attaining a diet sum score between 0 and 8 points; 11% had a low diet score (0–3 points), 54% had a moderate diet score (4–5 points), and 35% had a high diet score (6–8 points).

A small positive correlation between diet score and parental education was observed (*r* = 0.29, *p* < 0.0001).

### Intervention

A mean (SD) number of 44.0 (4.0) study meals were served to each child during the intervention. The meals had a mean (SD) concentration of 0.21 (0.15) mg/g EPA + DHA in the meat group and 15.2 (14.2) mg/g EPA + DHA in the fish group. Each meal had a mean weight of 71.1 (10.4) g. The children in the meat group had a higher mean (SD) total intake of meat (2675 (850) g) than the children in the fish group had of fish (2070 (978) g) (*p* < 0.0001). The food safety aspects of the intervention were evaluated (Additional file 1: Methods), and none of the participants exceeded 20% of the tolerable weekly intake (TWI) for dioxin and dioxin-like polychlorinated biphenyls (PCBs) from the study meals.

Changes in biochemical parameters from pre- to post-intervention are shown in Table 2. Children in the fish group had higher increased levels of EPA and DHA (milligrams/gram and percentage) from pre- to post-intervention compared to the meat group (Table 2). There were no differences in change of s-25(OH)D_3_ and UIC between the intervention groups, while there was a difference in s-ferritin, with a decrease in the fish group and no change in the meat group.

**Table 2:** Biological parameters measured at pre- and post-intervention and mean change

	Fish group (*n* = 105)					Meat group (*n* = 113)					
Biological parameters	No., *n*	Pre Mean (SD)	Post Mean (SD)	Change Mean (SD)	*p* ^a^	No., *n*	Pre Mean (SD)	Post Mean (SD)	Change Mean (SD)	*p* ^a^	*p* ^b^
Sum FA, mg/g	95	2.22 (0.30)	2.22 (0.27)	0.00 (0.40)	0.10	103	2.20 (0.24)	2.24 (0.24)	0.04 (0.26)	0.13	0.39
Sum SFA, mg/g	95	0.85 (0.10)	0.85 (0.09)	0.00 (0.14)	0.93	103	0.84 (0.08)	0.86 (0.10)	0.02 (0.10)	0.015	0.15
Sum MUFA, mg/g	95	0.45 (0.13)	0.44 (0.06)	−0.01 (0.14)	0.53	103	0.44 (0.09)	0.44 (0.07)	0.00 (0.09)	0.90	0.55
Sum PUFA, mg/g	95	0.84 (0.10)	0.85 (0.12)	0.01 (0.15)	0.41	103	0.83 (0.12)	0.85 (0.10)	0.02 (0.14)	0.21	0.82
LA, 18:2n-6, mg/g	95	0.25 (0.04)	0.26 (0.06)	0.01 (0.07)	0.39	103	0.25 (0.07)	0.25 (0.04)	0.00 (0.07)	0.59	0.33
AA, 20:4n-6, mg/g	95	0.29 (0.04)	0.28 (0.04)	−0.01 (0.05)	0.0290	103	0.29 (0.05)	0.30 (0.04)	0.01 (0.05)	0.0151	0.0011
EPA, 20:5n-3, mg/g	95	0.02 (0.01)	0.03 (0.01)	0.01 (0.01)	< 0.0001	103	0.02 (0.01)	0.02 (0.01)	0.00 (0.01)	0.38	< 0.0001
DPA, 22:5n-3, mg/g	95	0.04 (0.01)	0.04 (0.01)	0.00 (0.01)	0.46	103	0.04 (0.01)	0.04 (0.01)	0.00 (0.01)	0.12	0.63
DHA, 22:6n-3, mg/g	95	0.14 (0.03)	0.16 (0.04)	0.02 (0.03)	< 0.0001	103	0.14 (0.03)	0.14 (0.03)	0.00 (0.03)	0.15	0.0038
EPA, 20:5n-3, %	95	0.93 (0.46)	1.22 (0.51)	0.29 (0.40)	< 0.0001	103	0.88 (0.36)	0.90 (0.43)	0.02 (0.35)	0.57	< 0.0001
DHA, 22:6n-3, %	95	6.46 (1.20)	7.15 (1.30)	0.69 (1.03)	< 0.0001	103	6.39 (1.10)	6.41 (1.12)	0.02 (1.05)	0.68	< 0.0001
Omega-3 index^c^	95	7.38 (1.56)	8.37 (1.69)	0.99 (1.30)	< 0.0001	103	7.27 (1.33)	7.34 (1.43)	0.07 (1.27)	0.58	< 0.0001
s-25(OH)D_3_, nmol/L	92	62.4 (14.2)	69.8 (18.0)	7.4 (15.6)	< 0.0001	103	60.0 (13.8)	65.8 (15.8)	5.8 (10.8)	< 0.0001	0.39
s-Ferritin, μg/L	84	33.0 (21.6)	26.8 (12.7)	−6.2 (18.2)	0.0024	94	28.3 (16.7)	30.8 (20.3)	2.5 (17.4)	0.16	0.0013
UIC, μg/L	96	160.7 (94.6)	143.5 (69.4)	−17.2 (87.0)	0.06	104	151.1 (95.1)	123.6 (62.9)	−27.5 (95.5)	0.0041	0.43

Abbreviations: *AA* arachidonic acid, *DHA* docosahexaenoic acid, *DPA* docosapentaenoic acid, *EPA* eicosapentaenoic acid, *FA* fatty acids, *LA* linoleic acid, *MUFA* monounsaturated fatty acids, *PUFA* polyunsaturated fatty acids, *SFA* saturated fatty acids, *SD* standard deviation, *UIC* urinary iodine concentration, *25(OH)D*_*3*_ 25-hydroxyvitamin D_3_

^a^*p* for comparison within the intervention groups, paired samples *t* test

^b^*p* for comparison between the intervention groups, independent samples *t* test

^c^The content of EPA and DHA expressed as percentage of total fatty acids. All fatty acids are measured in red blood cells

The children in the fish group increased their mean (SD) body weight from 20.0 (3.3) kg to 20.8 (3.9) kg and the children in the meat group from 20.2 (3.0) kg to 21.0 (2.9) kg from pre- to post-intervention (*p* = 0.66).

### Main analyses

In the main analyses, there were no differences in change in WPPSI-III raw scores from pre- to post-intervention between the intervention groups for the total scale, the sub-scales, or on the sub-test level (Table 3). For the 9-HPT, the children in the fish group had a slightly better improvement for the non-dominant hand than the meat group (−4.5 s, 95% confidence interval (CI) –5.3, −3.2 vs −2.7 s, 95% CI −3.8, −1.7, *p* = 0.0470). No effect was observed for the dominant hand (Table 4).

**Table 3:** Predicted change in Wechsler Preschool and Primary Scale of Intelligence, 3rd edition (WPPSI-III) raw scores after study meals with fish (*n* = 105) and meat (*n* = 113)

	Main analyses					Sub-analyses			
				Models adjusted for					
				Pre-score, age^b^		Pre-score, age, compliance^**c**^		Pre-score, age, compliance, interaction treatment*compliance^d^	
Intervention	Pre Mean (SD)	Post Mean (SD)	*p* ^a^	Change Mean (95% CI)	*p*	Change Mean (95% CI)	*p*	Change Mean (95% CI)	*p*
WPPSI-III									
Total raw score									
Fish	145.3 (35.0)	162.7 (36.2)	< 0.0001	17.7 (14.8 to 20.7)	0.97	20.4 (17.5 to 23.3)	0.0060	21.9 (19.4 to 24.5)	
Meat	141.7 (33.5)	159.8 (33.1)	< 0.0001	17.8 (15.0 to 20.6)		15.2 (12.4 to 18.0)		17.2 (14.7 to 19.8)	
Model fit^e^							< 0.0001		< 0.0001
Verbal raw score									
Fish	56.6 (13.4)	60.4 (14.2)	< 0.0001	3.8 (2.6 to 5.0)	0.59	4.7 (3.6 to 5.8)	0.11	4.9 (3.8 to 6.1)	
Meat	55.9 (12.7)	60.2 (12.5)	< 0.0001	4.3 (3.1 to 5.4)		3.4 (2.4 to 4.5)		3.7 (2.6 to 4.8)	
Model fit^e^					0.0061		< 0.0001		0.072
Information									
Fish	23.8 (3.2)	24.8 (3.0)	< 0.0001	1.0 (0.6 to 1.4)	0.63	1.1 (0.7 to 1.4)	0.90	1.1 (0.7 to 1.4)	
Meat	23.5 (3.2)	24.7 (3.0)	< 0.0001	1.1 (0.8 to 1.5)		1.0 (0.7 to 1.4)		1.0 (0.7 to 1.4)	
Model fit^e^							0.067		0.95
Vocabulary									
Fish	18.0 (6.5)	19.2 (6.9)	0.015	1.1 (0.3 to 1.9)	0.99	1.7 (0.9 to 2.4)	0.0468	1.7 (1.0 to 2.5)	
Meat	18.4 (6.1)	19.5 (5.9)	0.006	1.1 (0.4 to 1.9)		6 (−0.1 to 1.3)		0.7 (−0.1 to 1.4)	
Model fit^e^							< 0.0001		0.36
Word Reasoning									
Fish	14.8 (5.2)	16.4 (5.5)	< 0.0001	1.8 (1.1 to 2.4)	0.50	2.0 (1.4 to 2.7)	0.54	2.2 (1.5 to 2.8)	
Meat	13.9 (4.9)	16.0 (5.1)	< 0.0001	2.1 (1.4 to 2.7)		1.8 (1.1 to 2.4)		1.9 (1.3 to 2.6)	
Model fit^e^							0.0001		0.036
Performance raw score									
Fish	51.7 (10.7)	57.3 (10.9)	< 0.0001	6.0 (4.7 to 7.3)	0.65	6.4 (5.2 to 7.7)	0.16	6.6 (5.4 to 7.9)	
Meat	49.2 (10.4)	55.2 (10.1)	< 0.0001	5.6 (4.4 to 6.8)		5.2 (4.0 to 6.4)		5.4 (4.2 to 6.6)	
Model fit^e^							0.0018		0.13
Block Design									
Fish	24.6 (3.6)	26.2 (3.4)	< 0.0001	1.7 (1.3 to 2.1)	0.068	1.8 (1.3 to 2.2)	0.0269	1.9 (1.4 to 2.3)	
Meat	24.3 (3.7)	25.5 (3.4)	< 0.0001	1.1 (0.7 to 1.6)		1.1 (0.6 to 1.5)		1.1 (0.7 to 1.6)	
Model fit^e^							0.14		0.15
Matrix Reasoning									
Fish	14.2 (4.8)	16.5 (4.7)	< 0.0001	2.5 (1.8 to 3.1)	0.52	2.5 (1.8 to 3.2)	0.48	2.5 (1.8 to 3.2)	
Meat	13.5 (4.3)	15.8 (3.9)	< 0.0001	2.2 (1.6 to 3.1)		2.2 (1.5to 2.8)		2.2 (1.5 to 2.8)	
Model fit^e^							0.72		0.91
Picture Concepts									
Fish	13.0 (5.0)	14.6 (5.1)	0.0015	2.1 (1.1 to 3.0)	0.91	2.4 (1.4 to 3.3)	0.26	2.4 (1.5 to 3.3)	
Meat	11.5 (5.4)	1.39 (5.2)	< 0.0001	2.0 (1.1 to 2.9)		1.7 (0.8 to 2.6)		1.8 (0.9 to 2.7)	
Model fit^e^							0.0011		0.45
Processing speed raw score									
Fish	37.0 (17.1)	45.0 (18.2)	< 0.0001	8.1 (5.9 to 10.3)	0.83	9.3 (7.1 to 11.4)	0.099	10.5 (8.4 to 12.5)	
Meat	36.6 (16.3)	44.4 (18.6)	< 0.0001	7.8 (5.7 to 9.9)		6.7 (4.6 to 8.8)		8.0 (6.0 to 10.0)	
Model fit^e^							< 0.0001		< 0.0001
Coding									
Fish	23.5 (12.0)	28.1 (12.9)	< 0.0001	4.5 (2.9 to 6.2)	0.58	5.4 (3.8 to 7.0)	0.41	6.2 (4.6 to 7.7)	
Meat	23.6 (10.6)	28.8 (12.4)	< 0.0001	5.2 (3.6 to 6.8)		4.4 (2.9 to 6.0)		5.3 (3.8 to 6.8)	
Model fit^e^							< 0.0001		< 0.0001
Symbol Search									
Fish	13.5 (7.1)	17.0 (6.9)	< 0.0001	3.6 (2.7 to 4.5)	0.12	3.9 (3.0 to 4.8)	0.0163	4.2 (3.3 to 5.1)	
Meat	13.0 (7.3)	15.6 (7.5)	< 0.0001	2.6 (1.7 to 3.5)		2.3 (1.4 to 3.2)		2.6 (1.7 to 3.5)	
Model fit^e^							0.0044		0.0027

Pre- and post-intervention data are given as mean (SD), change as mean (95% CI)

Abbreviations: *CI* confidence interval, *SD* standard deviation

^a^Paired samples *t* test for comparison of individual pre- and post-intervention values within each intervention group

^b^Linear mixed effect model adjusted for pre-intervention score and age

^c^Linear mixed effect model adjusted for pre-intervention score, age, and compliance (amount of fish/meat consumed)

^d^Linear mixed effect model adjusted for pre-intervention score, age, and interaction between treatment (intervention group) and compliance, mean values

^e^Likelihood ratio test to compare the goodness of fit to the previous model

A random intercept for kindergarten was included in all linear mixed model analyses

**Table 4:** Predicted change in 9-Hole Peg Test (9-HPT) seconds after study meals with meat (*n* = 113) or fish (*n* = 105)

	Main analyses					Sub-analyses			
				Models adjusted for					
				Pre-score, age^b^		Pre-score, age, compliance^c^		Pre-score, age, compliance, interaction treatment*compliance^d^	
Intervention	Pre Mean (SD)	Post Mean (SD)	*p* ^a^	Change Mean (95% CI)	*p*	Change Mean (95% CI)	*p*	Change Mean (95% CI)	*p*
9-HPT									
Dominant hand									
Fish	30.6 (7.8)	27.9 (6.1)	< 0.0001	−2.7 (−3.6 to −1.8)	0.19	−2.8 (−3.7 to −1.9)	0.09	−2.9 (−3.8 to −1.9)	
Meat	30.5 (7.6)	28.7 (6.1)	0.0043	−1.8 (−2.7 to −1.0)		− 1.7 (−2.6 to −0.8)		− 1.8 (−2.7 to −0.8)	
Model fit^e^							0.19		0.65
Non-dominant hand									
Fish	37.0 (10.3)	32.7 (7.6)	< 0.0001	−4.2 (−5.3 to −3.2)	0.0470	−4.5 (−5.6 to −3.4)	0.0110	−4.8 (−5.9 to −3.6)	
Meat	36.6 (8.9)	34.0 (7.4)	0.0003	−2.7 (−3.8 to −1.7)		− 2.5 (−3.5 to −1.4)		−2.8 (−3.9 to −1.7)	
Model fit^e^							0.0046		0.0027

Pre- and post-intervention data are given as mean (SD), change as mean (95% CI)

Abbreviations: *CI* confidence interval, *SD* standard deviation

^a^Paired samples *t* test for comparison of individual pre- and post-intervention values within each intervention group

^b^Linear mixed effect model adjusted for pre-intervention score and age

^c^Linear mixed effect model adjusted for pre-intervention score, age, and compliance (amount of fish/meat consumed)

^d^Linear mixed effect model adjusted for pre-intervention score, age, and interaction between treatment (intervention group) and compliance, mean values

^e^Likelihood ratio test to compare the goodness of fit to the previous model

A random intercept for kindergarten was included in all linear mixed model analyses

### Sub-analyses

In the sub-analyses, after adjusting for dietary compliance, the mean WPPSI-III total raw score improved more in the fish (20.4, 95% CI 17.5–23.3) compared to the meat group (15.2, 95% CI 12.4–18.0, *p* = 0.0060) (Table 3). No significant findings were revealed for the three sub-scales, but effects were evident in three of the sub-tests, where fatty fish was associated with improved performance on the Vocabulary, Block Design, and Symbol Search sub-tests of the WPPSI-III (Table 3). In the sub-analyses the improvement in the 9-HPT non-dominant hand remained stronger in the fish compared to the meat group, but still no effect was found for the dominant hand (Table 4).

**Fig. 2:**
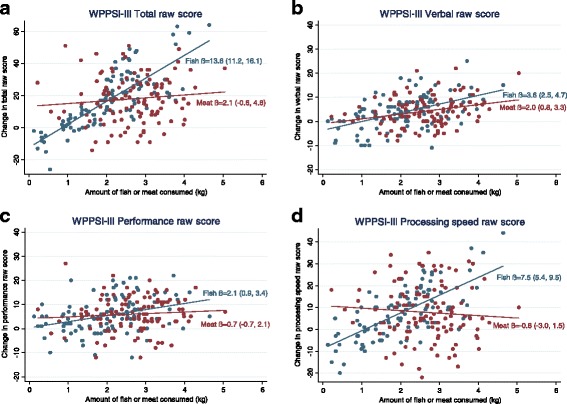
Scatter plots of changes in the Wechsler Preschool and Primary Scale of Intelligence, 3rd edition (WPPSI-III) from pre- to post-intervention vs amount fish (*blue*)/meat (*red*) consumed in kilograms (kg) (dietary compliance). The estimated regression line from an adjusted mixed effect model including the interaction between intervention and compliance, together with regression coefficient (β) and a 95% confidence interval are presented. Panel **a** shows WPPSI-III Total raw score, **b** Verbal raw score, **c** Performance raw score, and **d** Processing speed raw score

There was an interaction between intervention group and dietary compliance, reflecting that the WPPSI-III total raw score increased by 1.2 points more per 100 g eaten foods in the fish compared to the meat group (*p* < 0.0001) (Table 3). A similar interaction effect was also evident regarding processing speed, where the Processing speed raw score increased 0.8 point more per 100 g eaten foods in the fish compared to the meat group (*p* < 0.0001). This interaction was present in both Processing speed sub-tests, Coding and Symbol Search. The results were similar in the 9-HPT non-dominant hand, where the score decreased 0.19 s more per 100 g eaten foods in the fish compared to the meat group (*p* < 0.0027). There were no interaction effects for the Verbal and Performance sub-scales, or the 9-HPT dominant hand. The associations between WPPSI-III scores, intervention group, and dietary compliance are illustrated in Fig. 2a–d.

DHA (milligrams/gram) in RBC was found to be a mediation factor for WPPSI-III total raw score, explaining 19.2% of the difference in change of the total raw score between the two intervention groups. No mediation effects were observed for the 9-HPT, other biochemical parameters, or FFQ data (data not shown).

## Discussion

In this RCT, cognitive function in preschool children after intake of fatty fish or meat was examined. The main analyses revealed no differences in general cognitive function measured by WPPSI-III between the fish and meat groups. For the 9-HPT measure of fine-motor coordination, the fish group improved slightly more compared to the meat group for the non-dominant hand, but not for the dominant hand. In the sub-analyses, we found higher improvements in the fish compared to the meat group, after adjusting for dietary compliance in the total WPPSI-III raw scores and in three of the eight sub-tests, as well as in the 9-HPT non-dominant hand. There were no differences in the three main WPPSI-III raw-score sub-scales, Verbal, Performance, and Processing speed or in the 9-HPT dominant hand. An interaction effect was found between intervention and dietary compliance on both the total score and the Processing speed sub-scale score, and in the sub-tests comprising the Processing speed sub-scale. A similar effect was observed for 9-HPT non-dominant hand. This reflects that the scores increased more with higher dietary compliance in the fish than the meat group. EPA and DHA showed significant increases in the fish group compared to the meat group, and DHA was a significant mediation factor for the WPSSI-III total scores.

Serving herring and mackerel to preschool children did not increase cognitive functioning per se in the present trial. To our knowledge, this is the first RCT involving fatty fish consumption and cognitive function in preschool children, and other studies are thus not directly comparable. It has been suggested that n-3 LC-PUFA supplementation to healthy children may yield smaller impacts than in trials including children with poorer nutritional status [4, 36]. Thus, a possible explanation for the lack of an effect of fatty fish in the main analyses in the present trial could be that the children were not deficient in micro- or macronutrients prior to the intervention, as reflected by the FFQ data and the diet score. The diet score indicates that the children consumed little fruit and vegetables, but the score on the seafood intake, which is the most important factor for this study, was relatively high, and almost 50% of the children consumed seafood according to the recommendation of two to three times weekly. In comparison, about 40% of adolescents adhered to the seafood recommendations in the study by Handeland et al. [28]. In addition, taken together, the overall diet score and results from the biological analyses show that the diet as well as the levels of n-3 LC-PUFA, vitamin D, iodine, and ferritin status were good in these children. Furthermore, it has been suggested that studies of dietary n-3 LC-PUFA’s effect on cognition should have an intervention period of at least 4 months [4]. Although the 4-month intervention period was sufficient to yield an increase in the children’s RBC marine fatty acids, it may take longer until this increase leads to improvements of complex cognitive abilities [8]. The positive effect of the intervention in the 9-HPT non-dominant hand should be interpreted with care, given that we did not find a beneficial effect on the dominant hand. The beneficial effect could be due to an increased potential for improvements, as the non-dominant hand is less trained. However, this effect would be expected to be similar in both intervention groups.

In the sub-analyses, the improvements in the total WPPSI-III score, three of the sub-tests, and the 9-HPT non-dominant hand could indicate that a certain amount of fatty fish must be consumed for a beneficial effect to occur. These results are supported by the significant interaction between the intervention groups and dietary compliance on both the total scores and in the Processing speed sub-scale, reflecting that the cognitive scores increase more with higher fish intake. Our findings are supported by results from observational studies in Dutch adolescents where higher fish intake was associated with higher scores on vocabulary tests and end-term scores [18]. Furthermore, in a large cohort of pregnant women, findings indicate that the lower the maternal seafood intake during pregnancy, the higher the risk of sub-optimum development of their children [20]. In the latter study, a weekly intake of 340 g seafood is suggested as a cut-off, where an intake of less than this amount during pregnancy is associated with an increased risk of adverse outcomes in the offspring. Through our results we cannot suggest a similar ideal dose of weekly seafood intake in preschoolers, but our findings indicate a dose-response relationship between WPPSI-III raw scores and amount of fish consumed, as shown in Fig. 2. IQ scores are known to be stable measures that are not easily changed within an individual [23]. Note, however, that the significant findings in the sub-analyses in the current study were relatively small, and thus the clinical implications of our findings are unknown. Measures on long-term daily life functioning and academic achievement could give a broader understanding of the impact of our findings for these children. The lower consumption in the fish compared to the meat group could indicate that the children did not consume enough fish to produce improvements in cognitive function, and that a longer treatment period could be needed. Adjusting for parental education and family income did not alter the estimates or the *p* values, indicating that the effects, after accounting for the amount consumed, are independent of the socioeconomic situation for the family. In addition, intake of fish beyond the meals in the kindergartens (background diet) and hair mercury levels was also taken into account with no significance for the results. High exposure of mercury levels can have a negative effect on neurodevelopment [37]; however, the concentrations in the study fish meals were relatively low (Additional file 1: Table S1).

We did not find a beneficial effect of fatty fish on the Verbal and Performance sub-scales and in Processing speed in the sub-analyses considering dietary compliance. There was, however, a small beneficial effect in three out of eight sub-tests across the three sub-scales. Since these three sub-tests are from separate domains, we cannot conclude on any specific effects of the fatty fish. The interaction effect in the Processing speed sub-scale and the connected processing speed sub-tests, Coding and Symbol search, as well as 9-HPT non-dominant hand should be noted. This effect suggests that the speed of processing and fine-motor coordination improve more in the fish compared to the meat group relative to the amount of fish or meat the children consumed. The Processing speed sub-scale measures the ability to quickly and correctly scan and discriminate simple visual information [21]. DHA is important for neural communication and may thus affect the speed of processing [2]. There is some support for the significance of n-3 LC-PUFA for processing speed in children [7]. Tests of processing speed have rarely been included as an outcome per se, and thus more studies with pure tests of processing speed would be required to study the link between n-3 LC-PUFA and speed of processing further [4]. Improved fine-motor coordination has also been observed in children affected by phenylketonuria, after supplementation with fish oil for a period of 3 months. The children had a low dietary intake of n-3 LC-PUFA prior to the supplementation trial [38].

Our results demonstrate increased EPA and DHA levels from pre- to post-intervention in the fish compared to the meat group. In the explorative analyses, we found DHA to be the only biochemical parameter that mediated the effect on cognition. This finding is supported by the plausibility of an impact of n-3 LC-PUFA and especially DHA on cognition, substantiated by evidence for potential mechanisms [8]. Our findings are comparable to the findings in a Danish school-based general meal intervention study, where 3 months of school meals resulted in increased EPA and DHA status. EPA and DHA status was positively associated with cognitive performance, suggesting that the n-3 LC-PUFA explained approximately 20% of the intervention effect on the cognitive scores in the 8- to 11-year-old children [39]. We did not observe any significant mediation effects of vitamin D or iodine. The amount of vitamin D and iodine was relatively low in the served fish (Additional file 1: Table S1). This may explain why we observed a relatively small increment in vitamin D levels, and sun exposure may be an explanation for the similar increment in both the intervention groups in vitamin D from pre- to post-intervention (Table 2). In previous research, positive associations between maternal vitamin D status and infants’ cognitive function have been observed [13, 14]. However, such findings are not supported in RCTs with vitamin D supplementation in children [40]. Iodine supplementation has been found to improve cognitive function in mildly iodine-deficient children [12]. Other nutrients, such as vitamin B12 [41] and choline [42], could also be of importance according to the literature, but they were not taken into account in this trial.

Although outside the scope of this study, the increment of the omega-3 index to more than 8% among children in the fish group may have a positive influence on other aspects of health such as protection from coronary heart disease later on in life. In adults, an omega-3 index ≥ 8% shows greatest protection, and ≤ 4% shows least protection from coronary heart disease [43].

The strengths of this trial include the intervention design where the testers were blinded to treatment condition, individual randomization of participants, low attrition rate, close monitoring and biomarker determination, detailed information on nutrient content and amount of fish and meat eaten from the study meals, as well as the comprehensive measures of cognitive function. Our WPPSI-III data demonstrate high inter-rater agreement and scores that are comparable to the Norwegian normative sample, giving support to the validity and reliability of the collected data. Our sample size was slightly lower than calculated; still we had 78% power to detect an effect size of 0.37. Inclusion of specific neuropsychological measures could have yielded findings in other cognitive domains that may be under the influence of the n-3 LC-PUFAs. Furthermore, a longer intervention period may have been necessary to demonstrate significant results on cognitive function, although our results do indicate that 4 months may be sufficient to demonstrate differences as long as the participants consume sufficient amounts of the intervention meals. There is a possibility of training effects due to the short time lap between the pre- and post-testing; however, both groups would then be expected to have similar improvements. Inclusion of a no-intervention control group would enable the investigation of the possible effect of having meals served for lunch in the kindergarten compared to not. Finally, we did not obtain information about who filled out the questionnaire on behalf of the children, and it is not necessarily the same caregiver who completed it at pre- and post-intervention, which may have led to information bias. Reporting their children’s food intake could also result in over-reporting of healthy and under-reporting of unhealthy food groups due to the perceived social desirability of an intake in accordance with the recommendations.

## Conclusions

In conclusion, no significant effects of serving fatty fish were found on cognitive functioning measured by WPPSI-III in the main analyses. In the sub-analyses, taking the amount of fish or meat the children consumed into account, a beneficial effect of fatty fish was found on cognition, and DHA explains some of this effect.

## Additional files

Additional file 1: **Methods.** Undesirable substances in the meat and fish from the study meals. Analyses of energy and nutrients in the meat and fish from the study meals. References. **Table S1.** Energy and nutrients in the meat and fish from the study meals. (PDF 416 kb)

Additional file 2: **Table S2.** Dietary intake (background diet) presented as number of meals per week at pre- and post- intervention and mean change. **Figure S1.** Percentages of the participants (*n* = 197) who comply with the different recommendations in the diet score at baseline. **Figure S2.** Overview of the percentage of participants (*n* = 197) with diet score sums from zero to eight points at baseline. (PDF 452 kb)

## Abbreviations

9-HPT: 9-Hole Peg Test
CI: Confidence interval
DHA: Docosahexaenoic acid
EPA: Eicosapentaenoic acid
FFQ: Food frequency questionnaire
n-3 LC-PUFAs: n-3 long chain polyunsaturated fatty acids
RBC: Red blood cell
RCT: Randomized controlled intervention trial
SD: Standard deviation
WPPSI-III: Wechsler Preschool and Primary Scale of Intelligence, 3rd edition
