# Supplementary material for: Fatty fish intake and cognitive function: FINS-KIDS, a randomized controlled trial in preschool children
Source: BMC Med. 2018 Mar 12;16:41. doi: 10.1186/s12916-018-1020-z (PMC5848440; doi:10.1186/s12916-018-1020-z)
Supplement: Supplementary file 1 — Methods. Undesirable substances in the meat and fish from the study meals. Analyses of energy and nutrients in the meat and fish from the study meals. References. Table S1. Energy and nutrients in the meat and fish from the study meals. (PDF 416 kb) [file 12916_2018_1020_MOESM1_ESM.pdf]

Methods

Undesirable substances in the meat and fish from the study meals

In the study meals, the mean (SD) levels of dioxin and dioxin-like PCBs (pg TEQ/g) were 0.4 (0.1) in the fish and 0.1 (0.1) in the meat. The tolerable weekly intake (TWI) for dioxin and dioxin-like PCBs is 14 pg TEQ/kg body weight/week according to Scientific Committee of Food (SCF) [1]. The intake of dioxin and dioxin-like PCBs in this study represents 20% and 7% of the TWI from fish and meat meals, respectively when considering participants with average weight (20.1 kg) consuming average portion weights of the fish and the meat consumed per week during the 16 study weeks.

Analyses of energy and nutrients in the meat and fish from the study meals

Energy content of the meals was determined in bomb calorimetry and the gross energy was calculated from the calorimeter heat capacity and the temperature rise in water following the manufacturer's instructions (Parr instruments, Moline, IL, USA).

Total fat content was determined after acid hydrolysis and extraction in n-heptan (EU-directive 84/4 EØF, L 15/28, 18·1·84, method B).

For protein, the total nitrogen content was determined after combustion using a Nitrogen-Analyser (Perkin Elmer, 2410 Ser. II, Norwalk, CT, USA) and converted to crude protein assuming that proteins contain 16% N [2].

Fatty acid composition of total lipids was determined by a gas liquid chromatographic (GLC) method after extraction with 2:1 chloroform: methanol and internal standard 19:0 methyl esther. The fatty acid composition was calculated using an integrator (Chromeleon 6·80, Dionex Corporation, California, USA), connected to the GLC and identification ascertained by standard a mixtures of methyl esters (Nu-Chek, Minnesota, USA).

Vitamin D was determined by a reverse phase High Performance Layer

Chromatography (HPLC) analysis, using UV-detection and quantification by the internal standard method as described by Horvli and Lie [3].

Iodine was determined by inductively coupled plasma mass spectrometry (ICP-MS) after TMAH extraction [4].

Mercury was determined by ICP-MS after pressure digestion including the microwave heating technique [5].

Dioxins and dioxin-like PCBs were analysed using GC/MS as described by Berntssen et al [6, 7].

Selection of certified reference material was performed with regard to the similarity in concentration and matrix to sample material analysed to assess the trueness and precision of the analytical method in use. All methods used are according to NS-EN-ISO 17025 and the laboratory at Institute of Marine Research are frequently participating in proficiency tests.

References

1. Scientific Committee of Food. Commision Regulation (EC) No 1881/2006 of 19 December 2006 setting maximum levels of certain contaminants in foodstuffs. 2006. http://eur-lex.europa.eu/eli/reg/2006/1881/oj
2. AOAC, Official Methods of Analysis. Crude Protein in Meat and Meat Products. Combustion Method 1995. http://www.aoac.org/aoac_prod_imis/AOAC/Publications/Official_Methods_of_Analysis/AOAC_Member/Pubs/OMA/AOAC_Official_Methods_of_Analysis.aspx
3. Horvlie O, Lie O. Determination of vitamin D3 in fish meals by HPLC. FiskdirSkr Ser Ernæring. 1994;6:163-75.
4. Julshamn K, Dahl L, Eckhoff K. Determination of iodine in seafood by inductively coupled plasma/mass spectrometry. J Aoac Int. 2001;84:1976-83.
5. Julshamn K, Maage A, Norli HS, Grobecker KH, Jorhem L, Fecher P. Determination of arsenic, cadmium, mercury, and lead by inductively coupled plasma/mass spectrometry in foods after pressure digestion: NMKL1 interlaboratory study. J Aoac Int. 2007;90:844-56.
6. Berntssen MHG, Giskegjerde TA, Rosenlund G, Torstensen BE, Lundebye AK. Predicting world health organization toxic equivalency factor dioxin and dioxin-like polychlorinated biphenyl levels in farmed Atlantic salmon (Salmo salar) based on known levels in feed. Environ Toxicol Chem. 2007;26:13-23.
7. Berntssen MHG, Julshamn K, Lundebye AK. Chemical contaminants in aquafeeds and Atlantic salmon (Salmo salar) following the use of traditional- versus alternative feed ingredients. Chemosphere. 2010;78:637-46

Table S1 Energy and nutrients in the meat and fish from the study meals

	Energy (KJ/g)	Protein (mg/g)	Total fat (mg/g)	LA (mg/g)	AA (mg/g)	EPA (mg/g)	DPA (mg/g)	DHA (mg/g)	Vit. D (µg/g)	Iodine (ug/g)	Hg (µg/g)	Dioxin+dl-PCB (pg TEQ/g)
Meat												
Chicken gratin	7.2 (0.7)	120 (29)	53 (15)	10.2 (0.4)	0.18 (0.04)	0.04 (0.01)	0.05 (0.01)	0.06 (0.02)	NA	0.05 (0.04)	<0.002	0.11 (0.04)
Chicken strips	8.7 (1.1)	300 (14)	46 (20)	11.2 (0.2)	0.54 (0.08)	0.09 (0.01)	0.16 (0.01)	0.32 (0.01)	NA	<0.01	0.003	0.08 (0.01)
Chicken pie	18.8 (5.4)	142 (26)	255 (21)	18.7 (0.4)	0.81 (0.02)	0.09 (0.01)	0.16 (0.02)	0.31 (0.04)	NA	0.11 (0.01)	<0.002	0.15 (0.04)
Chicken bowls	7.7	128	76	11.1	0.45	0.03	0.06	0.06	NA	0.05	<0.002	0.10
Lamb bowls	17.2 (4.0)	185 (7)	245 (21)	4.1 (0.5)	0.40 (0.04)	0.14 (0.02)	0.33 (0.03)	0.05	NA	<0.02	<0.002	0.22
Cattle meatloaf	14.5 (3.7)	110 (12)	172 (15)	1.9 (0.1)	0.19 (0.03)	0.04	0.10 (0.03)	<0.01	<0.01	0.039 (0.003)	<0.002	0.17 (0.12)
Cattle patties	11.7 (4.2)	141 (11)	130 (29)	2.0 (0.1)	0.28 (0.04)	0.07 (0.01)	0.18 (0.02)	0.02 (0.01)	<0.01	0.037 (0.003)	<0.002	0.10 (0.02)
Cattle meatbolls	8.5	141	88	14.1	0.66	0.03	0.09	0.09	NA	0.04	<0.002	0.08
Cattle meat bowls	10.7	123	160	1.9	0.24	0.06	0.18	0.01	<0.01	0.04	<0.002	0.11
Average	12.0 (4.8)	153 (59)	138 (78)	7.5 (6.1)	0.40 (0.22)	0.07 (0.04)	0.15 (0.09)	0.14 (0.14)	<0.01	0.05 (0.03)	<0.002	0.13 (0.06)
Fish - herring												
Herring bowls - deep fried	13.3 (4.2)	148 (2)	135 (6)	35.4 (31.4)	0.21 (0.04)	2.15 (0.35)	0.34 (0.08)	4.00 (0.32)	0.09 (0.02)	0.078 (0.004)	0.031 (0.004)	0.45 (0.07)
Herring bowls - boiled	6.4 (0.5)	140 (4)	55 (5)	1.5 (0.2)	0.22 (0.05)	2.07 (0.39	0.35 (0.06)	3.93 (0.64)	0.10 (0.01)	0.08 (0.01)	0.035 (0.001)	0.47 (0.03)
Herring cakes	6.9 (0.2)	166 (9)	55 (2)	0.9 (0.1)	0.24 (0.02)	2.35 (0.11)	0.38 (0.01)	4.73 (0.34)	0.10 (0.02)	0.09 (0.01)	0.038 (0.002)	0.51 (0.03)
Herring pudding	5.9 (0.3)	135 (0)	49 (3)	0.83 (0.01)	0.18 (0.01)	1.63 (0.07)	0.27 (0.01)	3.19 (0.11)	0.08 (0.01)	0.10 (0.01)	0.030 (0.004)	0.42 (0.05)
Average	7.8 (3.0)	152 (15)	68 (33)	7.3 (17.1)	0.22 (0.03)	2.14 (0.33)	0.35 (0.06)	4.18 (0.67)	0.09 (0.02)	0.09 (0.01)	0.035 (0.004)	0.47 (0.05)
Fish - mackerel												
Mackerel gratin	8.7 (0.9)	95 (7)	129 (20)	7.0 (4.0)	0.57 (0.24)	6.69 (2.44)	1.18 (0.47)	11.17 (4.43)	<0.01	0.34 (0.07)	0.010 (0.003)	0.22 (0.05)

Mackerel sticks	26.3	185	365	27.2	1.54	17.02	2.94	28.01	0.05	0.12	0.034	0.59
	(9.2)	(21)	(35)	(3.5)	(0.11)	(0.59)	(0.23)	(2.26)	(0.01)	(0.01)	(0.006)	(0.10)
Mackerel pie	21.8	136	310	13.3	0.93	7.01	1.26	12.48	0.02	0.12	0.011	0.28
	(5.1)	(4)	(14)	(0.1)	(0.02)	(0.50)	(0.07)	(0.54)		(0.01)	(0.001)	(0.03)
Average	17.5	132	248	14.6	0.95	9.73	1.70	16.36	0.02	0.21	0.017	0.35
	(9.4)	(41)	(115)	(9.5)	(0.46)	(5.18)	(0.89)	(8.43)	(0.02)	(0.13)	(0.012)	(0.18)

Values indicate mean (SD)

Abbreviations: AA, arachidonic acids; DHA, docosahexaenoic acids; dl-PCB, dioxin-like polychlorinated biphenyls; DPA, docosapentaenoic acid; EPA, eicosapentaenoic acids; Hg, mercury; LA, linoleic acid; NA, not analyzed; SD, standard deviation

Single analysis of cattle meatbolls, meat bowls and chicken bowls. The same accessories were served in both intervention groups for each meal and it changed between cooked and fried potatoes, pasta, noodles, Ratatouille, couscous, Cauliflower, celery, raw and cooked carrots, Foccacia, white and wholegrain bread, tomato sauce, brown sauce. Grapeseed- and rapeseed oil were used for frying herring bowls and mackerel sticks
