# Supplementary material for: Fatty fish intake and cognitive function: FINS-KIDS, a randomized controlled trial in preschool children
Source: BMC Med. 2018 Mar 12;16:41. doi: 10.1186/s12916-018-1020-z (PMC5848440; doi:10.1186/s12916-018-1020-z)
Supplement: Supplementary file 2 — Table S2. Dietary intake (background diet) presented as number of meals per week at pre- and post- intervention and mean change. Figure S1. Percentages of the participants (n = 197) who comply with the different recommendations in the diet score at baseline. Figure S2. Overview of the percentage of participants (n = 197) with diet score sums from zero to eight points at baseline. (PDF 452 kb) [file 12916_2018_1020_MOESM2_ESM.pdf]

Table S2 Dietary intake (background diet) presented as number of meals per week pre- and post-intervention and mean change

Portions per week	Fish group (*n* = 105)					Meat group (*n* = 113)					
	No., *n*	Pre	Post	Change	*P*[a]	No., *n*	Pre	Post	Change	*P*[a]	*P*[b]
Seafood as dinner											
Seafood (all)	84	1.8 (0.9)	1.7 (0.9)	-0.1 (0.9)	0.22	90	1.6 (0.9)	1.6 (0.9)	0.0 (0.8)	0.93	0.32
Salmon/trout	84	0.6 (0.5)	0.5 (0.4)	-0.1 (0.5)	0.10	90	0.5 (0.5)	0.6 (0.6)	0.0 (0.5)	0.82	0.21
Mackerel	84	0.1 (0.2)	0.1 (0.4)	0.1 (0.3)	0.09	90	0.1 (0.3)	0.1 (0.4)	0.0 (0.3)	0.58	0.49
Herring	84	0.0 (0.1)	0.0 (0.2)	0.0 (0.3)	0.48	90	0.0 (0.0)	0.0 (0.0)	0.0 (0.0)	0.53	0.54
Halibut	84	0.1 (0.2)	0.1 (0.2)	0.0 (0.2)	0.78	90	0.0 (0.1)	0.1 (0.1)	0.0 (0.1)	0.16	0.53
Cod	84	0.4 (0.4)	0.4 (0.4)	0.0 (0.4)	0.38	90	0.4 (0.4)	0.4 (0.4)	0.0 (0.4)	0.62	0.74
Saith	84	0.2 (0.3)	0.2 (0.3)	0.0 (0.4)	0.72	90	0.2 (0.2)	0.2 (0.2)	0.0 (0.3)	0.83	0.83
Other lean fish	84	0.1 (0.2)	0.1 (0.2)	0.0 (0.2)	0.71	90	0.1 (0.2)	0.1 (0.1)	0.0 (0.2)	0.77	0.92
Sushi	84	0.0 (0.1)	0.0 (0.0)	0.0 (0.0)	0.75	90	0.0 (0.0)	0.0 (0.0)	0.0 (0.0)	0.67	0.62
Shellfish	84	0.1 (0.1)	0.1 (0.1)	0.0 (0.2)	0.71	90	0.0 (0.1)	0.1 (0.1)	0.0 (0.1)	0.94	0.81
Fish cakes	84	0.3 (0.2)	0.2 (0.2)	0.0 (0.2)	0.15	90	0.2 (0.2)	0.2 (0.2)	0.0 (0.2)	0.69	0.19
Fish sticks	84	0.2 (0.2)	0.1 (0.2)	0.0 (0.2)	0.26	90	0.1 (0.2)	0.1 (0.1)	0.0 (0.2)	0.10	0.73
Other fish products	84	0.2 (0.2)	0.2 (0.2)	0.0 (0.2)	0.67	90	0.2 (0.2)	0.1 (0.2)	0.0 (0.2)	0.55	0.86
Red meat as dinner	84	2.6 (0.8)	2.4 (0.9)	-0.2 (0.2)	0.0107	90	2.3 (1.0)	2.4 (0.9)	0.1 (1.1)	0.36	0.0227
Chicken as dinner	84	1.4 (0.9)	1.4 (0.9)	0.0 (0.8)	0.90	90	1.1 (0.9)	1.1 (0.8)	-0.1 (0.7)	0.49	0.72
Seafood as bread spread											
Seafood bread spread (all)	84	1.5 (1.5)	1.4 (1.5)	-0.1 (1.1)	0.48	90	1.2 (1.3)	1.0 (1.3)	-0.2 (1.1)	0.10	0.53
Mackerel in tomato	84	0.8 (1.0)	0.8 (1.1)	0.00 (0.8)	0.97	90	0.8 (1.1)	0.7 (1.1)	-0.1 (1.0)	0.55	0.63
Sardines	84	0.0 (0.2)	0.0 (0.1)	0.0 (0.1)	0.55	90	0.0 (0.1)	0.1 (0.2)	0.0 (0.2)	0.10	0.09
Other fatty fish	84	0.1 (0.3)	0.1 (0.2)	0.0 (0.3)	0.29	90	0.1 (0.3)	0.1 (0.3)	0.0 (0.3)	0.97	0.42
Tuna	84	0.0 (0.1)	0.0 (0.1)	0.0 (0.1)	0.42	90	0.0 (0.0)	0.0 (0.1)	0.0 (0.1)	0.07	0.11
Caviar	84	0.9 (1.3)	0.8 (1.2)	0.0 (1.1)	0.81	90	0.7 (1.1)	0.6 (1.0)	0.0 (0.9)	0.73	0.97
Fish pate	84	0.0 (0.0)	0.1 (0.3)	0.1 (0.3)	0.11	90	0.0 (0.2)	0.1 (0.6)	0.1 (0.5)	0.39	0.91
Egg	84	1.7 (1.1)	1.6 (1.2)	0.0 (0.8)	0.63	90	1.4 (1.2)	1.3 (1.0)	0.0 (0.8)	0.74	0.91
Milk and Dairy products	84	12.4 (7.0)	11.9 (7.2)	-0.5 (6.6)	0.50	90	12.2 (7.1)	11.3 (7.0)	-0.9 (7.2)	0.22	0.67
Fruit/vegetables											
Fruit/berries	84	9.6 (5.1)	8.5 (5.1)	-1.1 (5.7)	0.08	90	9.9 (5.4)	9.3 (5.6)	-0.6 (5.4)	0.31	0.54
Vegetables	84	7.3 (4.5)	7.6 (4.9)	0.3 (3.6)	0.54	90	6.5 (3.2)	6.7 (4.1)	0.2 (3.8)	0.57	0.98

Juice (no added sugar)	84	3.9 (3.7)	4.3 (4.4)	0.4 (2.9)	0.22	90	3.9 (4.1)	3.7 (4.3)	-0.2 (3.1)	0.65	0.24
Smoothie	84	1.1 (1.9)	1.2 (1.4)	0.1 (1.7)	0.46	90	1.0 (1.6)	1.0 (0.4)	-0.1 (1.5)	0.77	0.44
Drinks											
Water	84	3.4 (1.6)	3.1 (1.7)	-0.23 (1.9)	0.19	90	3.3 (1.7)	3.3 (1.7)	0.1 (1.6)	0.71	0.21
Diet Soda	84	0.1 (0.1)	0.1 (0.3)	0.0 (0.2)	0.44	90	0.1 (0.1)	0.1 (1.0)	0.0 (0.1)	0.26	0.72
Soda/ice tea (with sugar)	84	0.1 (0.1)	0.1 (0.1)	0.0 (0.1)	0.93	90	0.1 (0.1)	0.1 (0.1)	0.0 (0.1)	0.62	0.78
Sweets	84	1.8 (0.7)	1.7 (0.7)	-0.1 (0.5)	0.07	90	1.6 (0.6)	1.6 (0.5)	0.0 (0.6)	1.00	0.22

Values indicate mean (SD)

[a]*P* for comparison within the intervention groups, paired sample t-test

[b]*P* for comparison between the intervention groups, independent sample t-test

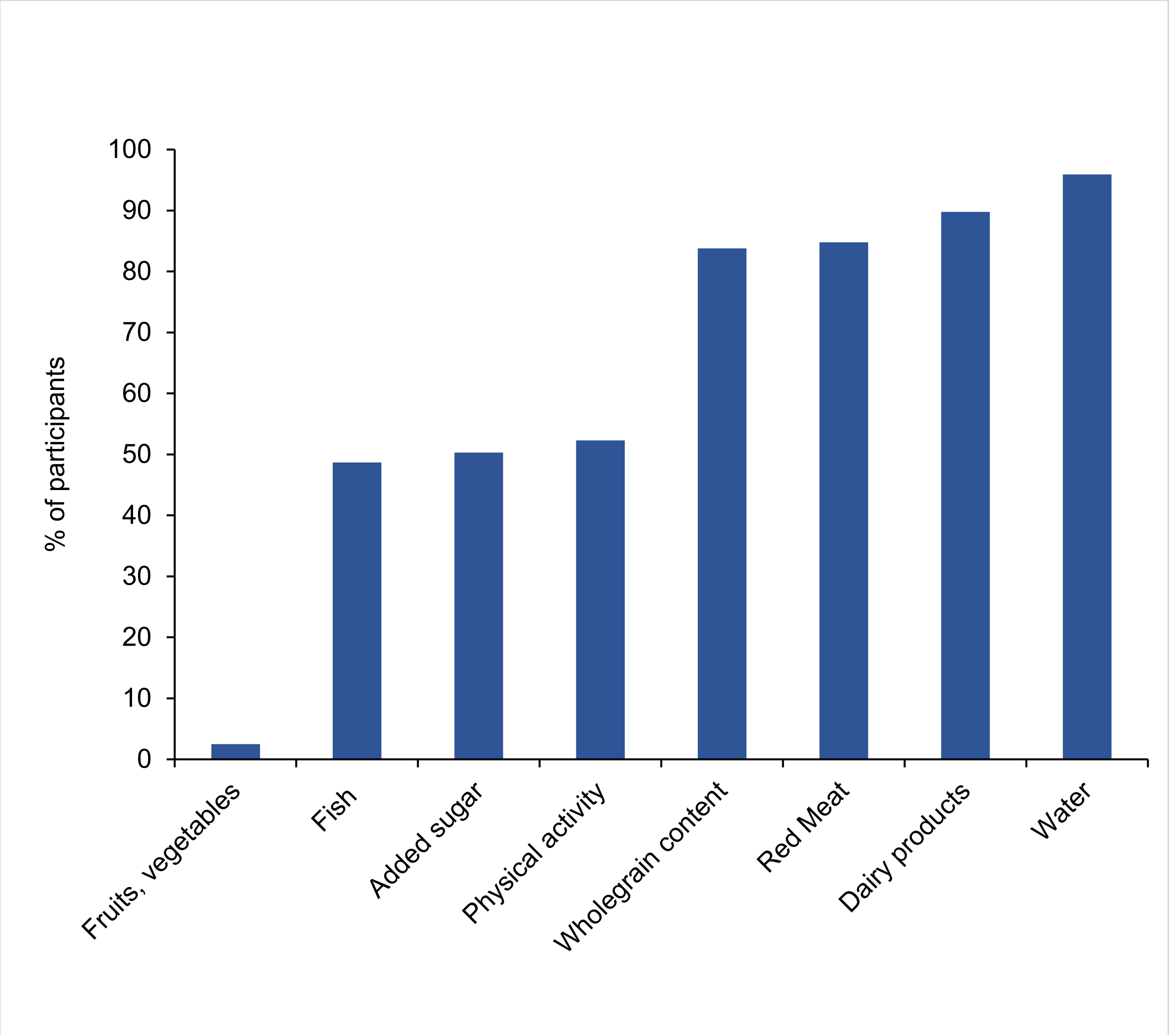

Figure S1 Percentages of the participants (*n* = 197) who comply with the different recommendations in the diet score at baseline

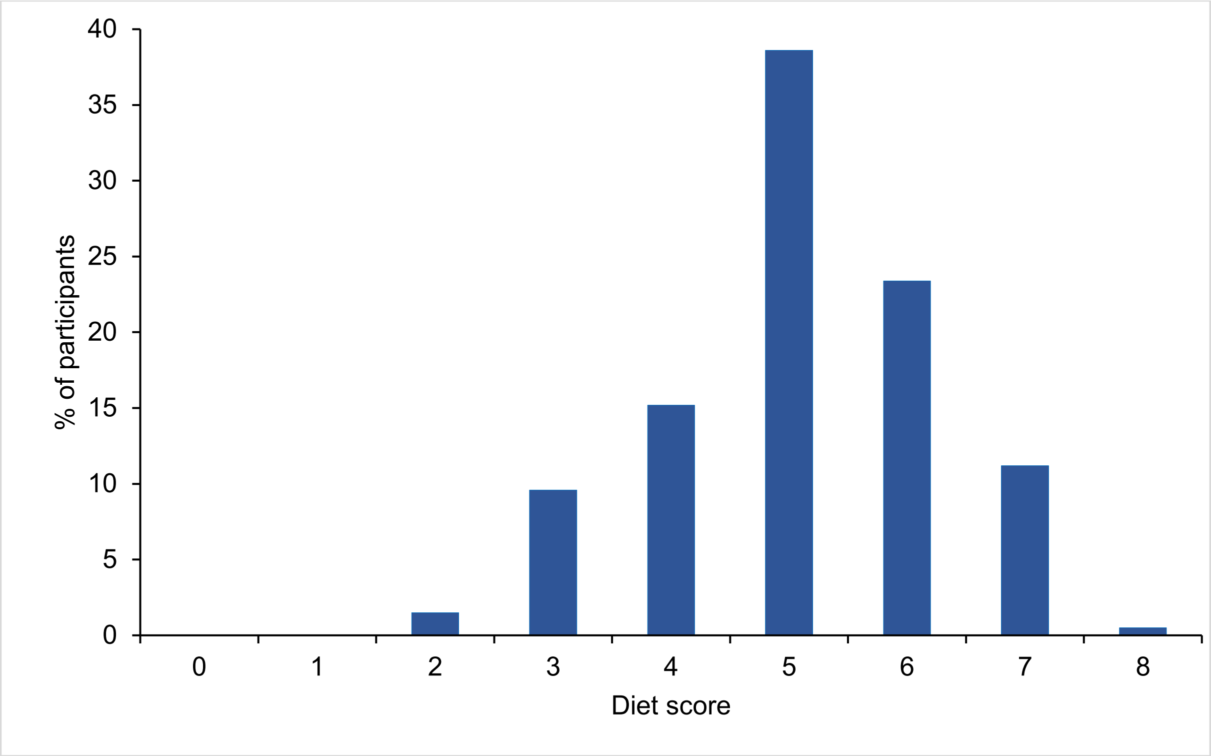

Figure S2 Overview of the percentage of participants (n = 197) with diet score sums from zero to eight points at baseline
